# Increased global placental DNA methylation levels are associated with gestational diabetes

**DOI:** 10.1186/s13148-016-0247-9

**Published:** 2016-07-26

**Authors:** C. Reichetzeder, S. E. Dwi Putra, T. Pfab, T. Slowinski, C. Neuber, B. Kleuser, B. Hocher

**Affiliations:** 1 Department of Experimental Nutritional Medicine, Institute of Nutritional Science, University of Potsdam, Arthur-Scheunert-Allee 114-116, Nuthetal, Potsdam 14558 Germany; 2 Department of Toxicology, Institute of Nutritional Science, University of Potsdam, Nuthetal, Germany; 3 Department of Nephrology, Campus Charité Mitte, University Hospital Charité, Berlin, Germany; 4 Center for Cardiovascular Research (CCR), Campus Charité Mitte, University Hospital Charité, Berlin, Germany; 5 Faculty of Biotechnology, University of Surabaya, Surabaya, Indonesia; 6 Diaverum Deutschland, Potsdam, Germany; 7 Institut für Laboratoriumsmedizin, Berlin, Germany; 8 Department of Basic Medicine, Medical College of Hunan Normal University, Changsha, China

**Keywords:** Placenta, Gestational diabetes, Insulin resistance, LC-MS/MS, Global DNA methylation, Epigenetics, Hypermethylation

## Abstract

**Background:**

Gestational diabetes mellitus (GDM) is associated with adverse pregnancy outcomes. It is known that GDM is associated with an altered placental function and changes in placental gene regulation. More recent studies demonstrated an involvement of epigenetic mechanisms. So far, the focus regarding placental epigenetic changes in GDM was set on gene-specific DNA methylation analyses. Studies that robustly investigated placental global DNA methylation are lacking. However, several studies showed that tissue-specific alterations in global DNA methylation are independently associated with type 2 diabetes. Thus, the aim of this study was to characterize global placental DNA methylation by robustly measuring placental DNA 5-methylcytosine (5mC) content and to examine whether differences in placental global DNA methylation are associated with GDM.

**Methods:**

Global DNA methylation was quantified by the current gold standard method, LC-MS/MS. In total, 1030 placental samples were analyzed in this single-center birth cohort study.

**Results:**

Mothers with GDM displayed a significantly increased global placental DNA methylation (3.22 ± 0.63 vs. 3.00 ± 0.46 %; *p* = 0.013; ±SD). Bivariate logistic regression showed a highly significant positive correlation between global placental DNA methylation and the presence of GDM (*p* = 0.0009). Quintile stratification according to placental DNA 5mC levels revealed that the frequency of GDM was evenly distributed in quintiles 1–4 (2.9–5.3 %), whereas the frequency in the fifth quintile was significantly higher (10.7 %; *p* = 0.003). Bivariate logistic models adjusted for maternal age, BMI, ethnicity, recurrent miscarriages, and familiar diabetes predisposition clearly demonstrated an independent association between global placental DNA hypermethylation and GDM. Furthermore, an ANCOVA model considering known predictors of DNA methylation substantiated an independent association between GDM and placental DNA methylation.

**Conclusions:**

This is the first study that employed a robust quantitative assessment of placental global DNA methylation in over a thousand placental samples. The study provides large scale evidence that placental global DNA hypermethylation is associated with GDM, independent of established risk factors.

## Background

Gestational diabetes mellitus (GDM) is defined as any degree of glucose intolerance with a first recognition during pregnancy [1]. In population based studies, the prevalence of GDM varies from 1 to 14 % with higher occurrences in specific populations [1, 2]. Ethnic disparities are existing, with Asians having the highest rate of GDM, followed by Hispanics, African Americans, and Caucasians [1]. GDM is associated with a markedly increased risk of adverse pregnancy outcome for both mother and infant. Mothers suffering from GDM have a higher risk for preeclampsia and cesarean delivery [3–5]. GDM pregnancies are associated with an increased perinatal morbidity and mortality, with an elevated risk for malformations, substantially higher rates of premature birth and neonatal hypoglycemia [3–5]. Newborns of pregnancies complicated by GDM show an increased risk to display altered growth patterns with increased neonatal body fat and a higher birth weight and ponderal index [3–5]. Evidence is accumulating that the intrauterine exposure to GDM also might contribute to long-lasting effects on the offspring by metabolic programming, leading to a higher disease susceptibility later in life [1]. Risk factors for GDM include age, a pre-pregnancy body mass index (BMI) >25.0 kg/m^2^, ethnic background, a history of GDM in previous pregnancies, glucosuria, a strong first-degree family history of type 2 diabetes or GDM, and a history of unexplained stillbirth or recurrent miscarriages [6, 7]. The placenta is the primary means of communication between mother and fetus and a target for maternal and/or fetal metabolic disturbances associated with GDM. Placentas of diabetic pregnancies show an increased placental to fetal ratio and are characterized by pathohistological findings [8]. The increased risk for adverse pregnancy outcome in GDM may be associated with these placental structural and functional changes. The molecular pathways behind this are incompletely understood so far. It is known that GDM is associated with altered placental gene regulation, and there is data indicating an involvement of epigenetic mechanisms [9–13]. So far, the focus regarding placental epigenetic changes in GDM was predominantly set on gene specific DNA methylation [10–13]. There are several studies that applied genome wide approaches, using the Illumina Infinium 450K BeadChips assay that measures CpG island methylation in about 99 % of all RefSeq genes [11–13]. However, such arrays only cover about 1.5 % of overall genomic CpGs, are biased towards the measurement of promoter methylation, and neglect other regions and functions of DNA methylation, thus cannot be regarded as a method to measure global DNA methylation [14, 15]. Furthermore, only about 1.5 % of the total genome sequence is comprised of protein encoding genes, while the remaining majority encompasses introns, repetitive elements, and other non-coding sequences [16]. Recent research has demonstrated important functions of DNA methylation in such non-coding genomic regions [14, 15, 17–19]. Although various sequencing methods are available for a detailed, site-specific analysis of global DNA methylation, these methods are very costly and time consuming, rendering them impracticable for DNA methylation analysis of large sample sizes [20]. Previous studies have demonstrated that the placenta is characterized by global DNA hypomethylation, usually a hallmark of various cancers. Next to global DNA hypomethylation, the placenta shares many features found in metastatic tumors, including rapid proliferation, invasiveness, and angiogenesis [21]. It was hypothesized that global DNA hypomethylation might support the unique functions of this organ [21–23]. Accordingly, alterations in the degree of global DNA methylation might be associated with altered placental function and disease. Until now, there are no large scale clinical studies that characterized global placental DNA methylation, assessed by an absolute quantification of the genomic 5-methylcytosine (5mC) content, in healthy and GDM afflicted pregnancies [24]. Thus, the aim of this study was to analyze global DNA methylation in over a thousand placental samples by the current gold standard method, LC-MS/MS, to truly asses the degree of global placental DNA methylation in uncomplicated pregnancies and to investigate whether GDM is associated with alterations in global placental DNA methylation.

## Results

**Fig. 1:**
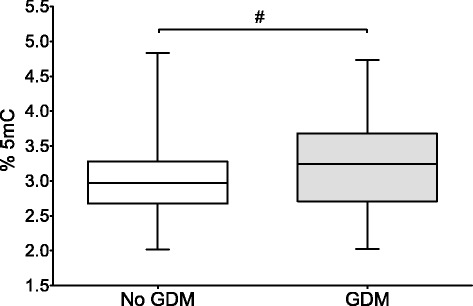
Box and whiskers plot displaying the mean global placental DNA 5mC content, according to the presence or absence of GDM. Mothers with GDM had significantly higher mean levels (3.00 %; min 2.02 %; max 4.84 %) of 5mC than mothers without GDM (3.22 %; min 2.03 % max 4.73 %); #: p=0.013

Table 1 displays detailed descriptive statistics of the 1030 mothers and their newborns and descriptive statistics of the study population grouped according to the presence or absence of GDM. Considering that patient recruitment was performed at a university clinic, the cohort did not display any uncommon features. The mean age of the mothers was 30.0 ± 5.9 years, and the mean BMI before pregnancy was 23.1 ± 4.5 kg/m^2^; 93.5 % of all mothers were of Caucasian, 0.9 % of African, 3.7 % of Asian, and 1.9 % of other ethnic background. Of all mothers, 5.4 % developed diabetes during pregnancy. The overall mean degree of placental DNA methylation was 3.01 ± 0.48 %. Data were normally distributed. Also shown in Table 1 are detailed descriptive statistics of the study population grouped according to the presence of GDM (*n* = 56) or no GDM (*n* = 974). Mothers with GDM had a significantly higher degree of placental global DNA methylation (3.22 ± 0.63 vs. 3.00 ± 0.46 %; *p* = 0.013; ±SD, Fig. 1), were older (31.9 ± 5.1 vs. 29.8 ± 5.9 years; *p* = 0.011; ±SD), and had higher HbA1c concentrations (58 ± 3.3 vs. 42 ± 6.6 mmol/mol; *p* < 0.0001; ±SD) and a significantly elevated BMI at the beginning and in the third trimester of pregnancy (24.8 ± 5.8 vs. 23.0 ± 4.4 kg/m^2^; *p* = 0.027; 29.6 ± 5.3 vs. 27.5 ± 4.4 kg/m^2^; *p* = 0.005). Newborns of GDM mothers had a significantly increased birth weight (3509.6 ± 742.5 vs. 3329.8 ± 627.1 g; *p* = 0.039), had an increased ponderal index (26.5 ± 3.0 vs. 25.4 ± 2.5; *p* = 0.002), and were more often delivered by c-section (40.0 vs 24.4 %; *p* = 0.030). Furthermore, the distribution of appropriate (AGA), small (SGA), and large (LGA) for gestational age newborns was significantly shifted towards a higher prevalence of LGA births in GDM mothers (62.5 % AGA/14.3 % SGA/23.2 % LGA vs. 78.6 % AGA/12.9 % SGA/8.6 % LGA; *p* = 0.001;). LGA was also associated with significantly increased placental DNA methylation (3.19 ± 0.55 %) compared to SGA (3.01 ± 0.43 %; *p* < 0.01) and AGA (2.99 ± 0.47 %; *p* < 0.001).

**Table 1:** Descriptive data of all mother/child pairs and grouped according to GDM

Maternal characteristics	All mothers (*n* = 1030)	Presence of GDM		
		No GDM (*n* = 974)	GDM (*n* = 56)	*p* value^a^
Maternal age, years	30.0 ± 5.9	29.8 ± 5.9	31.9 ± 5.1	0.011
Ethnicity (Caucasian, non-Caucasian), %	93.5/6.5	93.5/6.5	92.9/7.1	0.842
Maternal height, cm	166.9 ± 7.0	167.0 ± 6.9	166.4 ± 8.4	0.637
Body mass index before pregnancy, kg/m^2^	23.1 ± 4.5	23.0 ± 4.4	24.8 ± 5.8	0.027
Body mass index 3rd trimester, kg/m^2^	27.6 ± 4.5	27.5 ± 4.4	29.6 ± 5.3	0.005
Hypertension before/during pregnancy, %	2.3/3.8	2.3/3.6	1.8/7.1	0.809/0.331
Diabetes mellitus before/during pregnancy, %	0.0/5.4	0.0/0.0	0.0/100.0	–
Diabetes in family, %	38.1	37.7	44.6	0.300
Mean systolic blood pressure 3rd trimester, mmHg	116.2 ± 11.1	116.3 ± 11.1	114.1 ± 10.9	0.159
Mean diastolic blood pressure 3rd trimester, mmHg	70.3 ± 7.5	70.4 ± 7.5	69.4 ± 8.4	0.345
Smoking before/during pregnancy, %	35.3/13.6	35.5/13.7	32.1/12.5	0.613/0.801
Placental DNA methylation, %	3.01 ± 0.48	3.00 ± 0.46	3.22 ± 0.63	0.013
Parity number ≤1/≥2, %	75.7/24.3	75.9/24.1	72.2/27.8	0.545
s/p 2 or more miscarriages, %	6.5	6.4	8.9	0.449
Twin gestations, %	2.1	2.1	3.6	0.445
Gestational age at delivery, weeks	38.7 ± 2.1	38.7 ± 2.1	38.4 ± 1.8	0.056
Preterm birth (<37 weeks), %	10.3	10.4	8.9	0.728
Birth weight, g	3339.6 ± 634.8	3329.8 ± 627.1	3509.6 ± 742.5	0.039
AGA/SGA/LGA, %	77.7/13.0/9.4	78.6/12.9/8.6	62.5/14.3/23.2	0.001
Birth length, cm	50.6 ± 3.2	50.6 ± 3.1	50.3 ± 5.0	0.626
Child head circumference, cm	34.7 ± 1.7	34.7 ± 1.7	34.6 ± 1.8	0.719
Ponderal index, kg/m^3^	25.5 ± 2.5	25.4 ± 2.5	26.5 ± 3.0	0.002
Mode of delivery (spontaneous, OVD, c-section; %)	67.1/7.4/25.5	67.9/7.7/24.4	56.4/3.6/40.0	0.030
Child sex, male/female, %	51.7/48.3	51.6/48.4	53.6/46.4	0.780
Apgar score at 5 min	9.3 ± 1.0	9.3 ± 1.0	9.3 ± 0.9	0.996
Apgar score at 10 min	9.6 ± 0.8	9.6 ± 0.8	9.5 ± 0.7	0.137
Maternal HbA1c, %	6.1 ± 0.7	58 ± 3.3	42 ± 6.6	<0.0001

Data are given as mean ± SD or percentage

^a^Comparison between “GDM” and “no GDM”

**Fig. 2:**
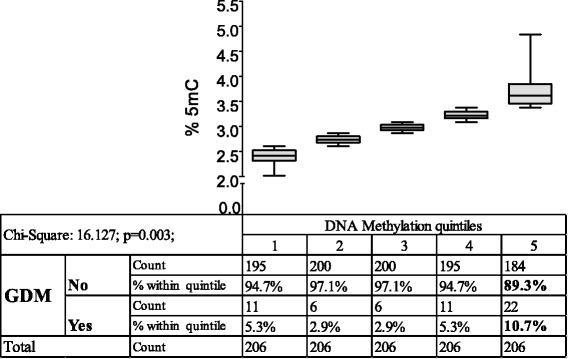
Crosstabulation of GDM and placental DNA methylation ranked in quintiles. Ranges of placental DNA 5mC are depicted on top each respective quintile as box and whisker plots (mean; min; max)

Bivariate logistic regression showed a highly significant correlation between placental DNA methylation and presence of GDM in an unadjusted model (Exp(B): 2.373; 95 % CI 1.425–3.954; *p* = 0.0009). To further categorize this relationship, quintiles of placental DNA methylation were generated (five groups of *n* = 206) and analyzed in crosstabs for the frequency distribution of gestational diabetes. Figure 2 shows a cross tabulation of placental DNA methylation ranked in quintiles and the occurrence of GDM. The frequency of GDM was evenly distributed in quintiles 1–4 with frequencies ranging between 2.9 and 5.3 %, only in the fifth quintile a significantly higher GDM frequency of 10.7 % was found (Pearson chi square 16.127; *p* = 0.003; Fig. 2). This observation was even more significant after re-stratification into two groups, the first consisting of placental DNA methylation quintiles 1–4 (*n* = 824) and the second of methylation quintile 5 (*n* = 206) (Pearson chi-square 13.766; *p* = 0.0002; Table 2).

Table 2 shows detailed descriptive statistics of the study population according to the abovementioned stratification into lower degrees of placental DNA methylation (quintiles 1–4; *n* = 824) versus high placental DNA methylation (quintile 5; *n* = 206). There were significant differences regarding the ethnic composition of the highest methylation group vs. methylation quintiles 1–4, with more non-Caucasians in the highest methylation group (9.7 vs. 5.7 %; *p* = 0.037). There was a significantly higher frequency of GDM in the highest methylation group (10.7 vs. 4.1 %; *p* = 0.0002). Moreover, a significant increase of LGA births was observed in the highest methylation group (73.0 % AGA, 12.7 % SGA, 14.2 % LGA vs. 78.8 % AGA, 13.0 % SGA, 8.2 % LGA; *p* = 0.028). Additionally, the Apgar score 10 min postnatally was significantly reduced in the highest methylation group (9.5 ± 0.8 vs. 9.6 ± 0.7; *p* = 0.011).

**Table 2:** Descriptive data of the mother/child pairs grouped according to the degree of placental DNA methylation

Maternal characteristics	Degree of placental DNA methylation		
	Quintiles 1–4	Quintile 5	*p* value^a^
	(*n* = 824)	(*n* = 206)	
Maternal age, years	29.9 ± 6.0	30.3 ± 5.5	0.407
Ethnicity (Caucasian, non-Caucasian), %	94.3/5.7	90.3/9.7	0.037
Maternal height, cm	167.0 ± 6.9	166.5 ± 7.3	0.365
Body mass index before pregnancy, kg/m^2^	23.2 ± 4.5	22.8 ± 4.4	0.094
Body mass index 3rd trimester, kg/m^2^	27.6 ± 4.5	27.4 ± 4.3	0.801
Hypertension before/during pregnancy, %	2.0/3.5	3.4/4.9	0.200/0.443
Diabetes mellitus during pregnancy, %	4.1	10.7	0.0002
Diabetes in family, %	38.1	38.2	0.964
Mean systolic blood pressure 3rd trimester, mmHg	116.4 ± 11.0	115.3 ± 11.5	0.202
Mean diastolic blood pressure 3rd trimester, mmHg	70.4 ± 7.5	70.0 ± 7.5	0.491
Smoking before/during pregnancy, %	35.4/13.3	34.6/14.7	0.828/0.635
Placental DNA methylation, %	2.84 ± 0.32	3.71 ± 0.34	<0.0001
Parity number <2/≥2, %	76.1/23.9	73.8/26.2	0.481
s/p 2 or more miscarriages, %	6.3	7.3	0.613
twin gestations, %	1.8	3.4	0.161
Gestational age at delivery, weeks	38.7 ± 2.1	38.8 ± 1.8	0.989
Preterm birth (<37 weeks), %	10.3	10.3	0.983
Birth weight, g	3334.8 ± 628.3	3358.5 ± 661.4	0.634
AGA/SGA/LGA, %	78.8/13.0/8.2	73.0/12.7/14.2	0.028
Birth lenght, cm	50.6 ± 3.1	50.5 ± 3.6	0.951
Child head circumference, cm	34.7 ± 1.7	34.6 ± 1.7	0.436
Ponderal index, kg/m3	25.5 ± 2.5	25.5 ± 2.7	0.943
Mode of delivery (spontaneous, OVD, c-section; %)	66.2/8.1/25.8	70.1/5.2/24.7	0.387
Child sex, male/female, %	52.3/47.7	49.8/50.2	0.522
Apgar score at 5 min	9.3 ± 1.0	9.2 ± 1.1	0.084
Apgar score at 10 min	9.6 ± 0.7	9.5 ± 0.8	0.011
Maternal HbA1c, m	43 ± 7.7	45 ± 8.7	0.257

Data are given as mean ± SD or percentage

^a^Comparison between “Quintiles 1–4” and “Quintile 5”

### Adjusted bivariate logistic regression models

To demonstrate that a high degree of placental DNA methylation is independently associated with GDM, adjusted bivariate logistic regression models were calculated. To account for confounding, the models were adjusted for available parameters known to be risk factors for GDM [6]. Risk factors included in the models were maternal age, BMI at the beginning of pregnancy, a history of recurrent (≥2) miscarriages (yes; no), ethnic background (Caucasian; non-Caucasian), and family history of type 2 diabetes (yes; no). Model A used these confounders together with placental DNA methylation as a continuous variable. In this model, placental DNA methylation was significantly associated with the presence of GDM ((exp)B = 2.408; 95 % CI 1.427–4.063; *p* = 0.001; Table 3). In model B, placental DNA methylation stratified into the before mentioned two groups of high placental DNA methylation (quintile 5; *n* = 206) and lower degrees of placental DNA methylation (quintiles 1–4; *n* = 824) was used. Grouped placental DNA methylation was associated with the presence of GDM even more significantly ((exp)B = 2.999; 95 % CI 1.691–5.316; *p* = 0.0002; Table 3).

**Table 3:** Model A: logistic regression investigating an association between placental DNA methylation and GDM; model B: logistic regression investigating an association between placental DNA methylation grouped into the fifth quintile (*n* = 206) versus quintiles 1–4 (*n* = 824) and GDM

Model A Constant −8.647 ± 1.589; *p* < 0.0001	B	S.E.	Sig.	Exp(B)	95 % CI for Exp(B)	
					Lower	Upper
Maternal age, years	0.061	0.026	0.017	1.063	1.011	1.118
BMI beginning of pregnancy, kg/m^2^	0.067	0.025	0.008	1.069	1.018	1.124
S/p 2 or more miscarriages, %	−0.128	0.502	0.798	0.880	0.329	2.351
Ethnicity (Caucasian, other)	−0.111	0.558	0.842	0.895	0.300	2.669
Diabetes in family (yes/no)	−0.318	0.289	0.271	0.728	0.413	1.281
Placental DNA methylation, %	0.879	0.267	0.001	2.408	1.427	4.063
Model B Constant −5.240 ± 1.275; *p* < 0.0001	B	S.E.	Sig.	Exp(B)	95 % CI for Exp(B)	
					Lower	Upper
Maternal age, years	0.061	0.026	0.017	1.063	1.011	1.118
BMI beginning of pregnancy, kg/m^2^	0.073	0.025	0.004	1.076	1.024	1.13
S/p 2 or more miscarriages, (%)	−0.143	0.501	0.775	0.867	0.325	2.314
Ethnicity (Caucasian, other)	−0.139	0.554	0.802	0.87	0.294	2.579
Diabetes in family (yes/no)	−0.314	0.289	0.277	0.731	0.415	1.286
Placental DNA methylation (grouped)	1.098	0.292	0.0002	2.999	1.691	5.316

### Adjusted ANCOVA model

To demonstrate that GDM is independently associated with global placental DNA methylation, an ANCOVA model was calculated and adjusted for predictors of DNA methylation evinced in previous methylation studies [25, 26]. These factors included smoking status before pregnancy (yes/no), ethnic background (Caucasian; non-Caucasian), sex of the child (male/female), and age of the mother. GDM was the strongest predictor of global placental DNA methylation (B = 0.215; 95 % CI 0.087–0.342; *p* = 0.001; Table 4) in this model. Additionally, the maternal smoking status before pregnancy was also significantly associated with global placental DNA methylation (B = −0.064; 95 % CI −0.126−0.002; *p* = 0.044; Table 4). Ethnicity of the mother, sex of the child, and maternal age showed no association with global placental DNA methylation (Table 4).

**Table 4:** ANCOVA analysis of the interaction between factors known to influence global DNA methylation, GDM, and global placental DNA methylation

Dependent variable: DNA methylation; *r* ^2^ = 0.016	*B*	S.E.	Power	Sig.	95 % CI for B	
					Lower	Upper
Intercept	3.06	0.091	1.000	<0.0001	2.881	3.239
GDM (yes/no)	0.215	0.065	0.911	0.001	0.087	0.342
Smoking before pregnancy (yes/no)	−0.064	0.032	0.521	0.044	−0.126	−0.002
Ethnicity (Caucasian, other)	0.112	0.061	0.457	0.064	−0.007	0.231
Sex of the child (male/female)	−0.032	0.03	0.191	0.279	−0.09	0.026
Maternal age, years	0.000	0.003	0.05	0.955	−0.005	0.005

## Discussion

The current study showed in a normally constituted cohort of delivering woman that there is a significant positive correlation between the degree of placental DNA methylation and GDM, i.e., higher levels of placental 5mC are associated with a higher frequency of GDM. Analyzing the mean levels of placental 5mC according to the presence or absence of GDM showed significantly higher mean values in GDM mothers. For a better understanding of the relationship between methylation and the risk for GDM, mothers were ranked in quintiles according to the degree of placental DNA methylation. This revealed a significantly elevated frequency of GDM in the fifth quintile. Calculating different bivariate logistic regression models with placental DNA methylation as continuous and categorical variable, adjusted for known risk factors of GDM, underlined an independent association between placental DNA hypermethylation and GDM. An ANCOVA model adjusted for known factors influencing DNA methylation further substantiated the independent association between global placental DNA hypermethylation and GDM.

One limitation of the current study is that no large data sets of continuous variables describing GDM were available, only categorical variables displaying the final diagnosis. Yet, measured term HbA1c concentrations in a subset of mothers (*n* = 94), which were significantly elevated in GDM mothers, reinforced a correct diagnosis of GDM. Another limitation of this study is the usage of placental tissue without focusing on a specific placental cell type. Given the large sample size (>1000 placenta samples), purification of specific placental cell types was not feasible. However, it was shown by MethylC-seq analysis that global methylation in whole rhesus placental tissue was almost identical compared to isolated rhesus trophoblast cell methylation with a correlation of 0.89 [21]. Next to the mentioned limitations, this study exhibits several strengths. To the best of our knowledge, it is by now the largest study of its kind analyzing placental DNA methylation and clinical readouts in 1030 mothers. Moreover, it is the first study that uses a robust quantitative assessment of placental global DNA methylation, employing the current gold standard LC-MS/MS [24]. All other studies investigating placental DNA methylation so far were either small, employed only semiquantitative methods of measuring global DNA methylation, or were focused on specific DNA methylation [9–12, 27]. Given the absence of studies with a comparable study design in regard to sample size, analyzed tissue, and the method of global DNA methylation assessment, interpretation of the results of the current study in context with available literature warrants caution.

Average levels of global placental DNA methylation were 3.00 ± 0.46 % in uncomplicated pregnancies. This is in agreement with another study analyzing global placental DNA methylation using HPLC, also demonstrating global DNA hypomethylation in comparison to average global DNA methylation levels observed in somatic tissues [28].

The current study demonstrated a positive association between GDM and global placental DNA methylation. Notwithstanding, a previous preliminary study by *Nomura* et al. showed a negative association between global placental DNA methylation and GDM [9]. However, as only 50 placenta samples were analyzed for global DNA methylation, the observed difference in results is most likely attributable to the ~20-fold smaller study size. Additionally, the seminquantitative luminometric methylation assay was used for the measurement of global DNA methylation, which was shown to be outperformed by other approaches of global DNA methylation assessment, especially regarding inter-assay comparability [9, 24, 29].

Two studies, investigating placental DNA methylation in GDM using the Illumina Infinium 450K BeadChips array, found a predominance of hypermethylation at methylation variable positions in GDM exposed placental samples [11, 12]. However, such arrays only cover about ~1.5 % of all genomic CpGs [14]. Therefore, the results of these studies have limited value in discussing the current observations [14].

Several clinical studies, investigating global DNA methylation in type 2 diabetes showed higher levels of global DNA methylation in different types of peripheral blood cells of diabetic patients [30–36]. Global methylation in these studies was assessed by LC/MS-MS [30, 33–35] or LC/MS-MS validated methods [31] or by measuring surrogate parameters of global DNA methylation like LINE-1 or Alu elements [32, 36]. The analysis of global DNA methylation in leukocytes is a well used method in epidemiologic studies to display associations between global DNA methylation and disease [35, 37, 38]. However, whether changes in global leukocyte DNA methylation associated with an insult (e.g., diabetes) also reflect aligned methylation changes in other tissues remains elusive. Nevertheless, a general hypothesis raised by Zhao et al., speculating that a putative link between global DNA hypermethylation and diabetes could be genomic instability, might also apply for findings of the current study [32]. The hypothesis is supported by findings of other studies [39–41], and there is ample amount of evidence that genomic instability is not only a hallmark of cancer but also associated with insulin resistance [42–44]. An underlying factor bearing implications for both genomic instability and DNA hypermethylation might be oxidative stress. Literature suggests that oxidative stress is involved in the development of metabolic diseases [45]. Diabetes itself is associated with increased oxidative stress, and it is well described that oxidative stress can induce DNA damage, another important factor in the development of diabetes [43, 45–47]. It was also shown that reactive oxygen species regulate the activity of DNA methyltransferases and catalyze the methylation of DNA [48, 49]. Moreover, it was demonstrated in animal models that an upregulation of the activity or the expression of DNA methyltransferases is observed in different types of diabetes [48, 50, 51]. Data from these recent animal experiments support a positive association between a diabetic metabolic state and global DNA methylation and additionally delineated putative mechanisms, connecting diabetes, oxidative stress, and global DNA hypermethylation [50, 51]. Zhong et al. have shown in a streptozotocin animal model of gestational diabetes that maternal diabetes induces an increased expression of DNA methyltransferases, increased DNA methyltransferase activity, and increased global DNA methylation (measured by MethylFlash Methylated DNA Quantification Kit which analyzes global 5mC content) in E8.75 embryos. Notably, treatment with the polyphenol epigallocatechin gallate, which was shown to exert anti-oxidative effects and to inhibit DNA methyltransferase activity, abrogated these changes [51–53].

## Conclusions

In summary, this study provides the first large scale evidence that placental global DNA hypermethylation is associated with GDM independent of established risk factors. The results of the study are in agreement with other studies; however, many of the comparable studies investigated DNA methylation in type 2 diabetes, in other organs than the placenta or by different methods of global DNA methylation assessment. Nevertheless, there is substantial evidence in literature from both clinical and preclinical experimental studies supporting an association between a diabetic metabolic state and global DNA hypermethylation. The results of the current study have to be confirmed using comparable methods, to substantiate our understanding of global DNA methylation involvement in disease. In general, a harmonization of applied methodology for the measurement of global DNA methylation is warranted. Differently designed studies will be needed to demonstrate a causal relationship between global DNA hypermethylation and GDM and to elucidate the still very elusive question whether aberrant global DNA methylation is involved in the pathogenesis of GDM or a consequence of this disease.

## Methods

### Ethics, consent, and permissions

The study was approved by the ethics committee of the university hospital Charité, Berlin, Germany. All clinical investigations were conducted according to the principles expressed in the Declaration of Helsinki. Written, informed consent was obtained from all partaking mothers prior to data collection.

### Clinical study

This observational all-comers study included 1063 mothers delivering at the obstetrics department of Campus Charité Mitte, Berlin, Germany. Structured interviews were performed with the partaking mothers. Eleven mothers with overt diabetes before pregnancy, nine cases of preeclampsia, and 13 cases with incomplete data of the most important variables and confounders were excluded from the statistical analyses. Given a potential overlap regarding pathophysiological processes leading to preeclampsia and GDM and the low sample number of preeclamptic placentas in the current study, it stood to reason to exclude mother with preeclampsia [9, 54]. In total, 1030 mothers were included into the statistical analyses. The majority of mothers (*n* = 963; 93.5 %) were of Caucasian ethnicity, while 67 mothers (6.5 %) had other ethnic backgrounds. Data from the “Mutterpass” documenting the results of follow ups during pregnancy were also collected. The following data were available for the dataset: age, ethnicity, body height, body mass index (BMI) at the beginning of pregnancy, body mass index (BMI) at the third trimester of pregnancy, parity, diabetes mellitus before or during pregnancy, family history of diabetes, incidence of hypertension before and during pregnancy, smoking before and during pregnancy, systolic and diastolic blood pressure measurements recorded during pregnancy, and in a small subset (*n* = 94) of mothers, maternal HbA1c levels at term. GDM was screened and assessed in all mothers according to the practice guideline of the German Diabetes Association (DGG) and the German Association for Gynaecology and Obstetrics (DGGG) [55]. Biometric data of the newborn were collected during the routine postnatal examination. Gestational age at delivery was based on the last menstrual period, anamnestically assessed during the first pregnancy examination. The following data of the newborn were added to the database: birth weight, birth length, head circumference, child sex, Apgar score 5 min postnatally and Apgar score 10 min postnatally. A standardized placenta sample—1 complete cotyledon (cross section of all layers) from similar locations—was collected and immediately frozen and stored at −20 °C. For the extraction of DNA a sample of chorionic villi was obtained from the whole cotyledon.

Data were analyzed using SPSS version 20.0 (SPSS, Inc, Chicago, IL, USA). Depending on normal distribution, unpaired *t* test or Mann-Whitney *U* test was used when comparing mean values of two groups. For the comparison of categorical variable distribution, chi-square test was used. Bivariate logistic regression was used to analyze the relationship of continuous and categorical data with a categorical outcome, to confirm relevant confounding variables that had an independent influence, and to adjust for these in different statistical models. To calculate associations between a continuous dependent variable and both categorical and continuous independent variables, ANCOVA models were used. Bar graphs were calculated and compiled with Graphpad Prism 5 (GraphPad Software Inc., La Jolla, California, USA). Probability values <0.05 were considered significant. The authors had full access to the data and take full responsibility for its integrity.

### Analysis of DNA methylation

DNA was extracted using a QIAamp DNA Mini Kit from Qiagen (Hilden, Germany) together with a RNase A digestion according to the manufacturer’s protocol. The concentration and quality of the RNA-free DNA solution were determined by a NanoDrop ND-1000 spectrophotometer. DNA hydrolysis was carried out using DNA Degradase Plus from Zymo Research (Freiburg, Germany). Briefly, 1 μg of genomic DNA was mixed with 2.5 μL 10× DNA Degradase Reaction Buffer, and 1 μL DNA Degradase Plus and filled up with water to a volume of 25 μL. DNA hydrolysis was stopped after 4 h at 37 °C by adding 75 μL of 0.1 % formic acid. Agarose gel electrophoresis using 200 ng of the digested DNA was employed in order to control the completeness of digestion. Seventy microliters of the hydrolised DNA samples were further diluted with 280 μL 0.1 % formic acid to yield a final concentration of 2 ng digested DNA/μL. DNA methylation was assessed by liquid chromatography-electrospray ionization/multi-stage mass spectrometry (LC-ESI/MS/MS) technique as described previously [56]. LC-ESI/MS/MS was performed with an Agilent 1200 series HPLC system connected to an Agilent 6530 Accurate-Mass Q-TOF instrument with Jet Stream-Interface (Waldbronn, Germany). For chromatographic separation a Waters (Milford, MA) X-BridgeTM C18 4.6 mm × 150 mm (3.5-μm particle size) protected by a Waters X-BridgeTM C18 4.6 mm × 20 mm guard column (5-μm particle size) was used. 0.1 % formic acid in water (solvent A) and 0.1 % formic acid in methanol (solvent B) were chosen as mobile phases. The linear gradient elution was 4–20 % of solvent B in 10 min at a constant flow rate of 0.5 mL/min. Fifty microliters of the diluted DNA hydrolysis samples, typically containing 100-ng digested DNA, were injected. The optimized ESI-MS/MS parameters in the positive ion mode were as follows: gas temperature, 250 °C; drying gas flow, 8 L/min; nebulizer pressure, 60 psig; sheat gas temperature, 300 °C; capillary voltage 4000 V; and collision energy 7 V for dC, 13 V for 5mdC, and 10 V for dG. Quantification was accomplished in selected reaction monitoring (SRM) mode by monitoring a transition pair of m/z 228.0979/112..0505 for dC, m/z 242..1135/126.0662 for 5mdC, and m/z 268.1040/152.0780 for dG, which was used as an internal standard for the measurement. The scan time was 333 ms for each pair. The deoxyribonucleosides 2′-deoxyguanosine (dG) monohydrate, 5-methyl-2′-deoxycytidine (5mdC) and 2′-deoxycytidine (dC) were purchased from ABCR (Karlsruhe, Germany). Hering sperm DNA was obtained from Sigma-Aldrich (Hamburg, Germany). Nuclease-free water used for DNA extraction was purchased from Roth (Karlsruhe, Germany). LC-MS-grade water, methanol, and formic acid were purchased from VWR international, Inc. (Dresden, Germany). The global level of DNA methylation was calculated as the percentage of DNA methylation as follows: DNA methylation % = 5-methyl-2′-deoxycytidine (5mdC)/[5-methyl-2′-deoxycytidine (5mdC) + 2′-deoxycytidine (dC)] × 100 %.

## Abbreviations

5mC, 5-methylcytosine; AGA, appropriate for gestational age; DGG German Diabetes Association; DGGG German Association for Gynaecology and Obstetrics; GDM, gestational diabetes mellitus; IADPSG, International Association of Diabetes in Pregnancy Study Group; LC-MS/MS, Liquid chromatography tandem mass spectrometry; LGA, large for gestational age; OVD, operative vaginal delivery; SGA, small for gestational age
